# Supplementary material for: Exploring shifts in values among urban Senegalese: The impact of global crises on social and cultural norms
Source: PLoS One. 2025 Jan 24;20(1):e0316162. doi: 10.1371/journal.pone.0316162 (PMC11759347; doi:10.1371/journal.pone.0316162)
Supplement: Attachment — Submitted filename: Response_to_Reviewers.docx [file pone.0316162.s002.docx]

June 15, 2024

Dr. Hamed Ahmadinia
Academic Editor

PLOS ONE

Dear Dr. Ahmadinia,

Thank you very much for reviewing our manuscript entitled “Study assesses changes in values of urban Senegalese during global crises.” We appreciate your time, effort, and providing insightful feedback on ways to strengthen our paper.

It is with great pleasure that we resubmit our manuscript for consideration. We have incorporated changes that reflect the detailed suggestions you have graciously provided. We have also ensured that all the journal requirements have been adequately fulfilled. We hope that our edits and the responses provided below satisfactorily address all the issues and concerns that you and the reviewers have noted.

To facilitate your review of our revisions, the following is a point-by-point response to the questions and comments delivered in your letter, dated April 10, 2024.

Thank you again for giving us the opportunity to strengthen our manuscript based on your constructive comments and suggestions.

Sincerely,

Yachiyo Tobita

University of Tsukuba

Ibaraki, Japan

**Point-by-point Response to the Editor and Reviewer**

- **Journal requirements**

1. When submitting your revision, we need you to address these additional requirements. Please ensure that your manuscript meets PLOS ONE’s style requirements, including those for file naming.

**RESPONSE**: We have changed the style to align it with the specifications of the PLOS ONE style.

2. You indicated that ethical approval was not necessary for initial 2018 study. Could you please provide confirmation from your institutional review board or research ethics committee (e.g., in the form of a letter or email correspondence) that ethics review was not necessary for this study? Please include a copy of the correspondence as an “Other” file. Additionally please include a complete copy of PLOS’ questionnaire on inclusivity in global research in your revised manuscript. Our policy for research in this area aims to improve transparency in the reporting of research performed outside of researchers’ own country or community. The policy applies to researchers who have travelled to a different country to conduct research, research with Indigenous populations or their lands, and research on cultural artefacts. The questionnaire can also be requested at the journal’s discretion for any other submissions, even if these conditions are not met.  Please find more information on the policy and a link to download a blank copy of the questionnaire here: <https://journals.plos.org/plosone/s/best-practices-in-research-reporting>.Please upload a completed version of your questionnaire as Supporting Information when you resubmit your manuscript.

**RESPONSE**: We apologize for the confusion caused by the lack of explanation regarding the ethical approval for the 2018 survey. We submitted all the necessary information, including the methodology and questionnaire, to the Senegalese Ministry of Higher Education and Innovation, which has an ethics review committee, as Gaston Berge University does not have an ethics committee. We obtained a research permit, including a comprehensive ethical approval, for the collaborative research project with Gaston Berger University. According to co-author Dr. Moussa Ndong, ethics approval is a part of the research approval process and serves as an official certification. We kindly ask you to verify the research permit, including a comprehensive ethical approval, letter issued by the Ministry of Higher Education and Innovation in 2018, Senegal, which we have already submitted as “Autorisation_Rechercher_2018. pdf”.

We have uploaded the PLOS’ questionnaire on inclusivity in global research as Supporting Information and reported in section Method.

3. We note that the grant information you provided in the ‘Funding Information’ and ‘Financial Disclosure’ sections do not match. When you resubmit, please ensure that you provide the correct grant numbers for the awards you received for your study in the ‘Funding Information’ section.

**RESPONSE**: Thank you for pointing this out. The grant organization, the Japan Society for the Promotion of Science, Overseas Challenge Program for Young Researcher indicated that only the support should be mentioned in the acknowledgements. Therefore, we changed our grant information as follow;

The study in 2018 was supported by public interest incorporated foundation, Urakami Foundation for Food and Food Culture Promotion (<https://www.urakamizaidan.or.jp/en.html>), grant number: 2017-30 (K.U.) and

public interest incorporated foundation, Food Culture Promotion and Ajinomoto Foundation for Dietary Culture (<https://www.syokubunka.or.jp/english/>),　grant number: 2017-01(Y.T. and K.U.). The study in 2022 was supported by ST-SPRING, Japan Science and Technology Agency (<https://www.jst.go.jp/EN/>),　grant number :JPMJSP2124(Y.T.). The funders had no role in study design, data collection and analysis, decision to publish, or preparation of the manuscript.

4. We note that your Data Availability Statement is currently as follows: [All relevant data are within the manuscript and its Supporting Information files.] Please confirm at this time whether or not your submission contains all raw data required to replicate the results of your study. Authors must share the “minimal data set” for their submission. PLOS defines the minimal data set to consist of the data required to replicate all study findings reported in the article, as well as related metadata and methods (<https://journals.plos.org/plosone/s/data-availability#loc-minimal-data-set-definition>).

For example, authors should submit the following data:

- The values behind the means, standard deviations and other measures reported;

- The values used to build graphs;

- The points extracted from images for analysis.

Authors do not need to submit their entire data set if only a portion of the data was used in the reported study.

If your submission does not contain these data, please either upload them as Supporting Information files or deposit them to a stable, public repository and provide us with the relevant URLs, DOIs, or accession numbers. For a list of recommended repositories, please see https://journals.plos.org/plosone/s/recommended-repositories.

If there are ethical or legal restrictions on sharing a de-identified data set, please explain them in detail (e.g., data contain potentially sensitive information, data are owned by a third-party organization, etc.) and who has imposed them (e.g., an ethics committee). Please also provide contact information for a data access committee, ethics committee, or other institutional body to which data requests may be sent. If data are owned by a third party, please indicate how others may request data access.

**RESPONSE**: Thank you for clarifying the requirements regarding the submission of the minimal dataset. The Ethics Committee of the University of Tsukuba prescribes that only statistical data can be provided when submitting papers or presenting at conferences. Therefore, we have uploaded the following statistical data and R script of Table 2, Table 3, and Figure 3 in the Supporting Information. We also changed the Data Availability Statement as follows;

Data Availability Statement : The relevant data and R script for analysis and visualization are available in the Support Information. The open-source datasets used in Table 4 is available at: [WVS Database (worldvaluessurvey.org)](https://www.worldvaluessurvey.org/WVSContents.jsp).

5. Please include your full ethics statement in the ‘Methods’ section of your manuscript file. In your statement, please include the full name of the IRB or ethics committee who approved or waived your study, as well as whether or not you obtained informed written or verbal consent. If consent was waived for your study, please include this information in your statement as well.

**RESPONSE**: Thank you for the comment. We have added the full ethics statement and obtained informed written consent in Method section (lines 283 to 287)

[Written consent was obtained from before the survey began. The comprehensive research permission including ethical approval for the 2018 survey was obtained from the Ministry of Higher Education Research and Innovation in Senegal (Approval No:0000303), and ethical approval for the 2022 survey was obtained from the Faculty of Life and Environmental Sciences of the University of Tsukuba(No:2022-2).]

**6.** Please upload a copy of Figure 1 and 2, to which you refer in your text on page 13 and 20. If the figure is no longer to be included as part of the submission please remove all reference to it within the text.

**RESPONSE**: We have uploaded Figure 1 and Figure 2.

7. Please upload a copy of Table S1, S2, S3 and S4 which you refer to in your text on page 38. Please upload them with the file type ‘Supporting Information’. Please ensure that each Supporting Information file has a legend listed in the manuscript after the references list.

**RESPONSE**: We have uploaded S1 file.(zip) as dataset and R script and added it in the manuscript after the reference list. Kindly refer our response about data availability in the journal requirement 4.

8. All supplementary figures and tables must be uploaded with the file type ‘Figure’. Please amend the file type to ‘Supporting Information’. Please ensure that each Supporting Information file has a legend listed in the manuscript after the references.

**RESPONSE**: We have no supplemental tables or figures.

9. Please review your reference list to ensure that it is complete and correct. If you have cited papers that have been retracted, please include the rationale for doing so in the manuscript text, or remove these references and replace them with relevant current references. Any changes to the reference list should be mentioned in the rebuttal letter that accompanies your revised manuscript. If you need to cite a retracted article, indicate the article’s retracted status in the References list and also include a citation and full reference for the retraction notice.

**RESPONSE**: We sincerely appreciate your comments. We have thoroughly reviewed our reference list to ensure its completeness and accuracy. To expand our discussion, we are adding 12 references to the revised manuscript as follow;

1. World Value Survey, Inglehart, R., C. Haerpfer, A. Moreno, C. Welzel, K. Kizilova, J. Diez-Medrano et al. (eds.). 2014. World Values Survey: Round Six - Country-Pooled Datafile Version
2. ANSD (Agence National de la Statistiqué et de la Démographie, Senegal) L’EHCVM est une des composantes principales du programme d’harmonisation et de modernisation des enquêtes sur les conditions de vie.
3. ANSD (Agence National de la Statistiqué et de la Démographie, Senegal) Projet a l’ecoute du Senegal 2014.
4. World Bank, Poverty & Equality Brief, Senegal 2022. Available from: Poverty and Equity Briefs (worldbank.org)
5. Inglehart, R., C. Haerpfer, A. Moreno, C. Welzel, K. Kizilova, J. Diez-Medrano et al. (eds.). 2014. World Values Survey: All Rounds - Country-Pooled Datafile Version.
6. Leijen, I., Van Herk, H., & Bardi, A. (2022). Individual and generational value change in an adult population, a 12-year longitudinal panel study. Scientific Reports.
7. Arieli, S., Grant, A. M., & Sagiv, L. (2014). Convincing Yourself to Care About Others: An Intervention for Enhancing Benevolence Values. Journal of Personality.
8. Lönnqvist, J.-E., Jasinskaja-Lahti, I., & Verkasalo, M. (2011). Personal Values Before and After Migration. Social Psychological and Personality Science.
9. Lönnqvist, J., Leikas, S., & Verkasalo, M. (2017). Value change in men and women entering parenthood: New mothers' value priorities shift towards Conservation values. Personality and Individual Differences.
10. Milfont, T. L., Milojev, P., & Sibley, C. G. (2016). Values Stability and Change in Adulthood. Personality and Social Psychology Bulletin.
11. African Business 2020, Cover story: Pandemic impact accelerates long-term trends for Orange.
12. GSMA The Mobile Economy Sub-Sahara Africa 2020.

- **Response to additional Editor Comments:**

**1:** Broader Implications: While your study adeptly addresses the immediate research questions, it is necessary to extend the discussion to include broader implications. Specifically, a deeper exploration of how these insights might be utilized by policymakers, health professionals, and other stakeholders is vital. This will enhance the practical utility of your findings and underscore their relevance in the current global context.

**RESPONSE**: Thank you for this valuable comment. We have extended the discussion to include the broader implications of our study and provide insights into how it might be utilized by policymakers, health professionals, and other stakeholders. Details have been included in our responses to the reviewer’s comment.

**2:** Temporary vs. Persistent Value Changes: The concept of whether value changes are temporary or persistent is intriguing and deserves more thorough examination. Your paper would benefit from a detailed discussion on the significance of both types of changes, offering insight into their respective implications within the context of your study.

**RESPONSE**: Thank you for this insightful comment. We have provided a detailed discussion of temporary and persistent value changes, highlighting the significance of both types of changes and their respective implications within the context of our study. Details have been included in our responses to the reviewer’s comment.

**3:** Sampling Size and Potential Biases: The disparity in sample sizes between your 2018 and 2022 surveys raises questions regarding comparability and potential biases. A more detailed explanation of how these differences were addressed in your analysis, as well as how the results can be generalized to the broader urban population in Senegal, is crucial for the credibility and reliability of your findings.

**REPONSE:** We have explained how we addressed issues of comparability and potential bias with respect to differences in sample size. Details have been included in our responses to the reviewer’s comment.

**4:**Consideration of Other External Factors: Your study currently focuses on the period between pre- and post-pandemic. However, it is important to consider other external variables that might have influenced societal value shifts during this timeframe. Factors such as cultural transformations, educational differences, and varying sample sizes should be thoroughly discussed to provide a more comprehensive understanding of the observed changes.

**RESPONSE**: Thank you for the suggestion. In the revised version, we have explored the external variables that may have influenced social value shifts between the pre- and post-pandemic periods. We discuss how the global crisis highlighted the inadequacy of the Senegalese social security system in the face of a dynamic social environment and the vulnerability of human life to various risks. Regarding educational differences, we explain how the differences in the number of years of education between the two groups are related to changes in values. Regarding the impact of sample size, we elucidate that the survey covered the broad urban population of Saint-Louis, including diverse neighborhoods ranging from fishing districts to commercial and working-class areas. To avoid potential bias, randomness was ensured in both surveys using a random walk method to select households. Please refer our responses to the reviewer’s comment for details.

- **Additional change of manuscript:**

Table 2 has been corrected because of a minor error in the calculation of household attribute data. These data were not used for statistical analyses and may not have been affected. Therefore, we have added additional sentences in the abstract and description of the respondent characteristics due to the change in values. In addition, we have changed the method of presenting cumulative values in the expenditure per day per capita comparison to follow the World Bank notation in Table 2.

To clarify the accuracy of the figures, the numbers in Table 3 have been also changed to three decimal places. The author's (YT) affiliation name has been changed to the correct affiliation name. It was the old affiliation name. Table 4 has been corrected minor changed. We sincerely apologize to the editors and reviewers for the inconvenience caused by these changes.

**The changed text are below:**

In lines 28 to 31 of the Abstract, we have changed the sentences in response to the correction of the calculation of the interaction rate.

[This study suggests that long-term economic insecurity and vulnerable social security have a greater impact on people’s values in Senegal than the threat of pandemic infection, since frequency of people’s social interactions increased compared to the pre-COVID-19 pandemic period.]

In the Discussion section, (line 434 to 437), we altered sentences in response of the correction of calculation in social interaction rate.

[In support of these data, the percentage of respondents in this survey who invited friends, relatives, and others for meals, indicating social interaction, increased after the pandemic compared to that before the pandemic.]

In the Discussion section, we have added new sentences regarding the impact of the global crisis on households. (line 498 to 501)

[Compared to the average consumption expenditure per person, it has decreased by 19.9% in 2022 compared to 2018. The 2022 survey indicated a distressing trend, with 29.4% of households living on less than US$3.65 per person per day, marking an increase of 11.1% from 2018 (Table.2).]

- **Point-by-point Response to the Reviewer**

Thank you very much for reviewing our manuscript entitled “Study assesses changes in values of urban Senegalese during global crises.” We appreciate the time and effort you dedicated to providing insightful feedback on ways to strengthen our paper.

**1:** The relevance of research in the current context is significant. Your study provides a crucial understanding of societal shifts during crises, which is highly relevant given the current global situation. While the research aligns well with the need for understanding societal dynamics in times of crisis, it would be beneficial to further articulate the broader implications of these findings. Specifically, how might these insights be utilized by policymakers, health professionals, or other stakeholders to better prepare for and respond to future crises?

**1: RESPONSE:** Thank you for this valuable comment. In the revised manuscript, we have provided a thorough discussion of the broader implications of our findings. We provide insights into how these results can be utilized by policymakers, health professionals, or other stakeholders to better prepare for and respond to future crises. Please refer to the Conclusion section (lines 597 to 607) as follows:

[The global crisis has highlighted the inadequacy of the Senegalese social security system in the face of a rapidly changing social environment and the vulnerability of human life to various risks. In response to crises such as COVID-19 and the Russia-Ukrainian war, people may have shown a shift in values that forced them to prioritize individual interests over the contribution to public interest as a whole. In addition, many African regions, including Senegal, are currently experiencing population growth and urbanization, increasingly linking them to the global context and making them more vulnerable than ever to the effects of global crises. Under these circumstances, there is a pressing need for the rapid development of health and social security systems, taking into account the high probability that mutual trust and cooperation between societies will be destabilized during a crisis.]

**2:** Temporary vs. persistent value changes: Your paper briefly mentions that individual values may revert to their original state over time. This point is particularly intriguing and warrants further exploration. Could you elaborate on the implications of temporary versus persistent value changes in your study? Are temporary value changes equally significant or is the focus just on enduring changes? Could you elaborate on the impacts of temporary value changes?

**2: RESPONSE:** Thank you for your thoughtful comment. Regarding persistent and temporary change in values, we have expanded the discussion as follows (lines 505 to 534):

[The results of this study involve a comparison between two different periods, and it would be difficult at this point to assess whether the change observed is temporary or persistent. Previous research has identified three main categories of value change. First, there is short-term change due to psychological experiments. Second, intra-individual value change results from external social events such as war, economic crisis, immigration, and pandemics, including life events such as attending school, marriage, becoming a parent, and retirement. Third, intra-individual value change is associated with aging. However, although individual values change significantly when confronted with major events, such as terrorism, financial crisis, and immigration, they slowly return to their original values over the long term. Although such change in values can be seen as an adaptation to new social changes or stress, the direction of the change in values depends on the individual’s inherent values and cultural context.

Intra-individual value change associated with aging is an overall change in values throughout life. For example, as people age over time and experience different events, their values increase the importance of conservation and self-transcendence, while openness to change decreases. However, recent research suggests that value differences vary by generation. Value differences associated with age differences include both generational value differences and internal changes over time. These value changes may influence the development of social values in the long run. Therefore, when considering the relationship between temporary and persistent changes in values, it is conceivable that a temporary change in values caused by a major social event could influence the value formation of a particular generation.

Furthermore, a generation that has experienced such a change in values may change over time due to age and experience, resulting in differences between other generations. However, these studies are limited to wealthy countries and need to be expanded. It is imperative to further examine whether the value changes observed in this study were temporary phenomena or persisted over time, taking into account factors such as regional and cultural differences, individual experiences with global crises, and socioeconomic conditions.]

**3:**Sampling size and potential biases: The disparity in sample sizes between the 2018 (60) and 2022 (200) surveys raises concerns about comparability and potential biases. The larger sample size in 2022 might lead to more nuanced insights and analyses that were not feasible with the smaller sample in 2018. How you have addressed these sampling differences in your analysis? and how you generalize your results to the broader urban population in Senegal?

**RESPONSE:** Thank you for the comment. To address potential bias and comparability in the sample size differences between the 2018 and 2022 surveys, we have added the following elucidation in the Survey Design section (lines 264 to 272) :

[Students from Gaston Berger University participated as investigators for both the 2018 and 2022 surveys, and the same procedures were used to conduct the interview. While the 2018 survey covered households living on the sandbank islands in the Senegal River as well as in commercial and working-class districts, the 2022 survey was expanded to cover larger districts. As a result, the survey covered a broad urban population of Saint-Louis city by including diverse neighborhoods ranging from fishery districts, commercial districts, and working-class neighborhoods. To avoid potential bias, randomness was ensured in both surveys by using a random walk method for selecting households.]

**RESPONSE:** The representativeness in the sample was also addressed by making a comparison with the demographic data obtained from the Senegalese Statistical Agency as follows (lines 306 to 313):

[The amount of daily consumption per person in the household was 1672 FCFA in 2018 and 1339 FCFA in 2022. According to the ANSD, the average daily per capita consumption expenditure in urban areas (including Dakar) is 1818 FCFA, while in rural areas, it is 1040 FCFA, which is a reasonable value given that the survey was conducted in a rural city. The number of household members per household was 8.9 in 2018 and 11.3 in 2022, which is generally similar to the 10.4 in urban reported by ANSD. This result indicates that the sample is representative of this study.]

**RESPONSE:** The statistical method for the identification of changes in values before and after the COVID-19 pandemic have been explained as follows (lines 359 to 363):

[Table 3 shows the results of statistical tests comparing the means of each value item before and after the pandemic. Notably, due to the different sample sizes in the 2018 and 2022 surveys, it is not possible to apply the conventional t-test, which assumes equal variances between samples. Instead, the two-tailed results of the Welch test are applied.]

**RESPONSE:** To generalize the results of this study to the broader urban population of Senegal, we did the following: Calculated the average per capita daily expenditure of the surveyed households in Saint-Louis City and compared it with urban and rural values in Senegal. Moreover, the daily per-capita expenditure of the households was classified according to World Bank indicators, and its impact on the global crisis was discussed (line 314 to 328).

[The amount of daily consumption expenditure per person is further examined based on the World Bank’s definition. The World Bank defines a daily consumption expenditure of US $2.15 per person as the international poverty line, US $3.65 or less as the lower middle-income class poverty line, and US$6.85 as the upper middle-income class poverty line. According to the World Bank, a daily consumption per person of less than US $2.15 is considered the international poverty line, which percentage was 9.3% for Senegal as a whole. Less than the lower middle-income class poverty line was 37.4%, and the upper middle-income class poverty line was 74.4%. However, the number of households below the international poverty line increased slightly from 3.33 % in 2018 to 3.95 % in 2022 when looking at the change between 2018 and 2022. Conversely, households below the lower middle-income class poverty line have increased from 18.33% (2018) to 29.38% (2022), which means that the number of households below the upper middle-income class poverty line has increased over the past four years from 58.33% to 76.84%. The results show an overall decline in daily household consumption.]

**RESPONSE:** In response to the comment that the larger sample size in 2022 allows for more nuanced insights and analysis, we described an increase in the social interaction rate (inviting friends, neighbors and other to sharing meals) from 23.16% to 33.9% (lines 302 to 305). We find this interesting in terms of behavior. However, we are currently working on another paper that examines the relationship between interaction behavior and value; therefore, we would prefer not to analyze it in depth in this paper.

**4:** Consideration of Other External Factors: This study captures the societal value shifts between the pre- and post-pandemic periods. However, it is critical to consider other intervening variables that may have influenced these changes across the four-year study period. For example, cultural transformations, educational differences, and sample size differences, could all have a significant impact. Could you elaborate on how your study considered these factors?

**RESPONSE**: Thank you for the suggestion. We have expanded on how other external variables that might have influenced societal value shifts during this timeframe and added our thoughts in lines 576 to 581 of the conclusion as follows:

[In the context of the COVID-19 pandemic, rapid urbanization led to the expansion of the mobile phone market and financial changes due to the spread of mobile payments. However, external shocks such as the COVID-19 pandemic and the Russia-Ukraine war have led to high levels of informal employment and high inflation in Senegal. The impact of these social changes on culture is expected to be significant.]

**RESPONSE:** Regarding the impact of educational differences and culture change, we have expanded the discussion as follows (lines 582 to 596):

[Considering the difference in years of education between the two groups in this study, it is conceivable that this educational disparity is related to the change in values. The differences in values toward the crisis may be the result of differences in the ease of gathering information, participation in COVID-19 sanitation efforts, and work opportunities. However, a comparison of years of education and employment rates by district in the 2022 survey shows that respondents living in districts closer to commercial areas tend to have higher employment rates, suggesting that years of education may not be related to work opportunities. In addition, although public education in Senegal is provided in French, information in the widely spoken Wolof language is readily available through the media and social networking sites, creating a social environment in which even the illiterate population can access a wide range of information. Therefore, the impact of differences in years of education on changes in values is likely to be minimal at this point. However, it would be premature to discuss the impact of cultural change on people’s values in detail. This requires careful examination after gathering more detailed information and reviewing previous studies from various perspectives.]

Regarding sample size differences, we have explained how to consider these differences in Response 3. Kindly refer to it.

Thank you again for giving us the opportunity to strengthen our manuscript based on your valuable comments and queries. We have worked hard to incorporate your feedback and hope that these revisions persuade you to accept our submission.
