# Supplementary material for: Exploring shifts in values among urban Senegalese: The impact of global crises on social and cultural norms
Source: PLoS One. 2025 Jan 24;20(1):e0316162. doi: 10.1371/journal.pone.0316162 (PMC11759347; doi:10.1371/journal.pone.0316162)
Supplement: Attachment — Submitted filename: 2nd-revised response to reviewrs.docx [file pone.0316162.s003.docx]

15, October 2024

Dr. Sadia Malik

Academic Editor

PLOS ONE

Dear Dr. Sadia Malik

We wish to re-submit the manuscript titled “Exploring shifts in values among urban Senegalese: The impact of global crises on social and cultural norms.” The manuscript ID is PONE-D-23-37707R2. Old title was “Study assesses changes in values of urban Senegalese during global crises”

We thank you and the reviewers for your thoughtful suggestions and insights. The manuscript has benefited from these insightful suggestions. I look forward to working with you and the reviewers to move this manuscript closer to publication in the PLOS ONE.

The manuscript has been rechecked and the necessary changes have been made in accordance with the reviewers’ suggestions. The responses to all comments have been prepared and given below.

To facilitate your review of our revisions, the following is a point-by-point response to the questions and comments delivered in your letter, 1 of August, 2024. We have tracked the changes within the manuscript.

Thank you for your consideration. I look forward to hearing from you.

Sincerely,

Yachiyo Tobita

Ph.D. student, Graduate School of Science and Technology, Degree program in Life and Earth Sciences, University of Tsukuba

1-1-1 Tennoudai, 305-8577 Ibaraki, Japan

Email: yachiyo.tobita@gmail.com

**Point-by-point Response to the Reviewer**

Comments from Reviewer 2

**ABSTRACT**

**Comment 1**: Abstract: somehow theoretical, try to enlarge with the present results from the article.

**RESPONSE:** Thank you for pointing this out. We agree with this comment. Therefore, we have enlarged with the present result in abstract section (line 23 to 32) as follow;

[Surveys were conducted in Saint-Louis, Senegal, in August-September 2018 and June-July 2022. The timing of these studies coincides with the onset of the COVID-19 pandemic in early 2020 and the outbreak of the Russia-Ukraine war in February 2022. The findings revealed a 19.9% decrease in the average monthly cost of living per capita between 2018 and 2022, attributed to the combined effects of rising food prices and unemployment. Furthermore, the proportion of households spending less than $3.50 per person per day—below the lower-middle-income class poverty line—increased by 11.05%. Our analysis indicates a decline in values such as benevolence, universalism, hedonism, and self-direction. In contrast, values related to power and achievement significantly increased following the pandemic.]

**INTRODUCTION**
**Comment 2**:[ Introduction: NOVELTY + results (better explanation)]

**RESPONSE:** : Thank you for this insightful comment. We have, accordingly, revised introduction to emphasize the novelty and results in introduction section ( line 63 to 64)

[Research has utilized quantitative value indicators to examine how external factors, such as global economic crises, influence changes in human values]

In line 81 to 102

[During this period, several global events unfolded, including the emergence of COVID-19, the outbreak of the Russia-Ukraine war, and the resulting global economic turbulence. By comparing values across these two time periods, we aim to shed light on how these historic global events impacted urban Senegalese values.

There is a notable lack of quantitative studies focusing on the detailed psychological changes among urban Senegalese populations. The novelty of this study lies in its quantitative assessment of changes in values during this period for a specific urban population in Africa, using a standardized value indicator that allows for comparison with other countries and time periods.

The structure of this paper is as follows: First, we provide an overview of Schwartz’s theory of values, which serves as the study’s theoretical framework. Subsequently, we review studies on individual changes due to COVID-19, using value indicators to highlight the novelty of our research. In the methodology section, we detail the original data collection process. While previous studies indicate that the pandemic led to an increased emphasis on conservative values, the shift in value orientation among urban Senegalese people demonstrated a somewhat different trend. The discussion section analyzes the psychological changes observed in light of our survey findings. Finally, in the conclusion section, we discuss the factors that influenced value changes in Senegalese people, address the limitations of this study, and suggest areas for future research. We believe that by assessing the changes in individual values observed in urban Senegal as a result of the global crises, this study will contribute meaningfully to the growing body of research in this area.]

**METHODOLOGY**

**Comment 3:** Methodology: why the authors use only these indicators into the model and only this country? Present some theoretical explanations for these indicators.

**RESPONSE:** Thank you for this valuable comment. One of the reasons for using this indicator is that we had the opportunity to conduct a comprehensive survey in 2018, prior to the onset of the pandemic and the war, which informed the direction of this study.

We have provided a theoretical explanation regarding the use of this indicator.

(in line 212 to 213)

[In this study, we assessed participants’ values using survey questions from the WVS Wave 6 (2014–2017), based on Schwartz’s ten dimensions value dimensions.]

(in line 224 to 230)

[The value indicators were developed as a standardized method for quantitatively measuring the psychological scales of individuals with varying cultural backgrounds. Numerous international comparative studies, including the WVS, have employed these indicators. The primary advantage of employing these indicators is their standardization, which facilitates future international or intertemporal comparisons. Notably, there is a dearth of value surveys focused on West African countries, and this study aims to contribute to advancing research in this area]

To explained regarding Best Worst Scaling reviews (in line 236-264)

[We used BWS, specifically Case 1, to evaluate respondents’ values. BWS is a survey method designed to measure people’s preferences or the relative importance they assign to multiple items. It has been widely applied in various academic fields in recent years, including health, psychology, and agricultural economics.

In a BWS survey, a subset of all items is presented to respondents, who then choose the most important (best) item and the least important (worst) item from that set. In addition, respondents select one attribute within each question as either the best or worst, making it easier for them to understand and engage with the question. BWS has also been used in surveys based on Schwartz’s theory of basic values. For example, Lee et al. quantitatively measured Schwartz’s ten value items using BWS and highlighted that traditional self-report ratings often suffer from the problem of respondents applying different evaluation criteria. BWS helps reduce this effect by providing a more structured comparison. According to Lee et al., BWS is an effective method for revealing people’s values because it is both more straightforward and relative than traditional methods, such as the Likert scale. Furthermore, Lee et al. conducted a BWS assessment of 19 value items, including new items such as animal welfare, in samples of adults from Australia and the US, demonstrating the utility and reliability of this approach. Daniel et al. used BWS to examine the impact of the pandemic on individual values, employing the counting approach to determine the relative importance of different factors. Similarly, Sneddon et al. used the BWS counting approach to explore changes in individual values related to the natural environment during the pandemic. The results of these studies suggest that the BWS is an effective approach for capturing changes in individual values. To design the survey, we used the support.BWS package in R Core Team version 4.2.2. to create BWS Case 1 questions. The question design followed a Balanced Incomplete Block Design (BIBD), implemented using the crossdes package. Table 2 shows the combinations of value items, each randomly displayed six times using BIBD. As shown in Figure 3, respondents were presented with 11 questions, each consisting of six items, and were asked to select one attribute from the six that was either “very much like me ” or “not at all like me.” ]

**Comment 4:** Methodology: the main problem of this study is the analysis. The sample of respondents is too small (60 first and 200 after). How the authors choose these people?

**RESPONSE:** You have raised an important point. While increasing the sample size to a more statistically significant number would have been more appropriate, the sample size in this study is limited. However, to ensure the representativeness of the sample, we have used random sampling techniques, which we believe addresses the concerns regarding participant selection. We have added a detailed explanation of the sampling method and the process for selecting respondents in the Methods section.

In line 289 to 292

[Africa’s demographic transition and rapid urbanization are significantly impacting the lives of its people. Given our emphasis on evolving individual values in urban Africa, we focused on Senegal, which has the highest urbanization rate in West Africa.]

In line 296 to 305

[The first survey took place before the pandemic, from late August to early September 2018. After discussions with local collaborators, nine representative districts in Saint-Louis were selected for the study. A random walk method was employed to select six to seven households per district, resulting in a sample of 60 households.

The second survey was conducted between the post-pandemic period and the Russia-Ukraine War, from early June to late July 2022, using the same random walk method. Of the 33 districts, 10 were selected using probability proportional to size sampling, based on census data provided by the Agence National de la Statistiqué et de la Démographie. A total of 20 households were selected from each district, yielding a total sample of 200 households]

**Comment 5**:Methodology: I recommend for authors to read and apply some very serious survey analysis (example: Chapman & Hall - Applied Survey Data Analysis CRC Statistics in the Social and Behavioral Sciences, 2nd Edition)

**RESPONSE:** Thank you for this comments. We agree with this and have incorporated your suggestion throughout the manuscript.

**FIGURES AND TABLES**
**Comment** **6**: Introduce at least five tables and five figures for the analysis

**RESPONSE:** We agree with this and have incorporated your suggestion throughout the

manuscript. To achieve a total of five tables and five figures, three additional figures and one additional table are incorporated.

The following tables and figures are presented:

Introduction section, line104

Figure 1 Change in import broken rice prices in Saint-Louis (2017-2022)

(In line 137)

Figure 2 Theoretical model of the structure of relationships among ten motivational types of value (Schwartz, 2012).

We add explanation about Figure 2 in line 126 to 127).

[As shown in Figure 2, Schwartz’s theory of basic values outlines ten core human value orientations, which are grouped into four higher-order dimensions ]

Figure 3 Example of choice set for Best Worst Scaling (in line 269)

Figure 4 Survey location in Senegal.(in line 332)

Figure 5 Standardized BW score in 2018 and 2022 surveys.(in line 393)

Table 1 Question for each value attribution based on Schwartz’s theory of basic values.(in line 232)

Table 2 A combination of number of displayed value items in questionnaire. (in line 266)

Table 3 Descriptive statistics of respondents in the 2018 and 2022 surveys.(in line 365)

Table 4 Result of Welch’s t-test.(in line 413)

Table 5 Comparison of values in francophone West African countries.(in line 483)

**ANALYSIS
Comment 7:** The analysis is very small and sketchy; enlarge this part of the article

**RESPONSE:** Thank you for pointing this out. We agree with this comment. Therefore, we have enlarged and explained in line 371 to 378.

[Based on the aggregated standardized BW scores from the 2018 and 2022 surveys, we compared the responses for each value item. Figure 5 displays the mean standardized scores for each value item. A positive score indicates that respondents consider the value important, while a negative score suggests the opposite. This comparison highlights respondents’ value tendencies over time. In both surveys, tradition—representing family and religious values— consistently ranked as the most important. Respondents also emphasized benevolence-caring, symbolizing kindness toward others, and conformity, reflecting social discipline. These values correspond to the conservation and self-transcendence dimensions of values.]

We add in line 400 to 406.

[Table 4 presents the results of statistical tests conducted to evaluate the differences in the mean values for each item between 2018 and 2022. Given the unequal sample sizes in the two surveys, a standard t-test assuming equal variances was not applicable. Instead, the Welch test was employed to account for these differences. A two-tailed test was conducted with the null hypothesis that there were no significant changes in the values of respondents between 2018 and 2022. If a significant difference in the mean values was found, the null hypothesis was rejected.]

In line 443 to 456, we enlarged this part and add subsection summary.

[Using the Welch test, we examined the changes in values and identified those with significant differences. The overall trend in values remained consistent across 2018 and 2022. Tradition (from the conservation group), along with benevolence-caring and benevolence-dependability (from the self-transcendence group), were identified as the most important values in both years. However, achievement and power (self-enhancement values), which were not deemed important in 2018, became more significant in 2022. Conversely, the importance of hedonism and self-direction (openness to change values) declined significantly.

The examination of changes in standardized BW scores across the two time points revealed several values with significant differences. Power and achievement, which reflect financial success and social status, showed a significant increase in scores. In contrast, self-direction and hedonism, values associated with creativity and personal enjoyment, experienced a decline. Additionally, scores for benevolence-caring, benevolence-dependability, and universalism—values tied to environmental and societal contributions—also decreased significantly.]

**Comment** **8**: Analysis: introduce a larger sample for respondents calculated according to the survey analysis.

**RESPONSE:** Thank you for your comments. You have raised an important point here. However, the dataset analyzed in the manuscript was obtained from the original survey conducted in 2018 and 2022. Consequently, it is not feasible to conduct further surveys using data from previous years. We believe that using these samples is a more appropriate approach given the unfeasibility of expanding the sample size of the dataset.

**Comment** **9**:Analysis: the survey MUST be on the same respondents in 2019 (before), 2020 (pandemic), 2022 (war) and 2024 after with the same questions. After that, analyze the answers before, during, and after

**RESPONSE:** Thank you for this suggestion. It would have been interesting to explore this aspect. However, due to the numerous restrictions that were in place during the period of the COIVD-19 pandemic and in the subsequent period, it would have been challenging to achieve this within the scope of our study. Furthermore, we would like to clarify that this is not an online survey. The two surveys were designed via face-to-face interviews conducted over five consecutive days to collect data. Due to the various restrictions imposed by the circumstances of the ongoing pandemic and the need to ensure the safety of our surveyors, we were unable to proceed with the survey, particularly during the 2020 and 2021 periods.

**DISCUSSION
Comment 10**: Discussions: at least 2 pages; separate from the conclusions

**RESPONSE**: Thank you for the suggestion. Actually, we have over two pages of previous manuscript. In the revised version, we have added sentences to provide further detail in the discussion.

We outlined the reasoning behind our analysis of values in Senegal in comparison to other West African countries, as detailed in line 459 to 464.

[This study aimed to quantitatively assess changes in the values of urban residents in Senegal during a global crisis. Previous research on values has demonstrated that cultural similarities often lead to common values tendencies. To validate this survey, we compared its results in Senegal with the WVS conducted in Mali and Burkina Faso, both member countries of the Economic Community of West African States (ECOWAS). This comparison helped us assess similarities, differences, and the robustness of the findings. ]

We enlarge the result of comparing value of Senegalese and Mali and Burkina Faso in line 468 to 472.

[Our survey included two dimensions, benevolence-caring and benevolence-dependability, covering a total of 11 value items. In contrast, the WVS surveys in Burkina Faso and Mali focused on the broader benevolence dimension, with 10 value items. Despite these slight differences, it was still possible to compare the values expressed across these surveys.

We also enlarge the result in line 473 to 481,

[As shown in Table 5, the values of benevolence under self-transcendence, and tradition and conformity under conservation, were consistently prioritized across the three countries. On the contrary, values such as stimulation, hedonism, and power were not highly preferred. This indicates a common trend in Senegal and other West African countries, where self-transcendence values (such as benevolence) and conservation values (such as tradition and social cohesion) are prioritized, while self-enhancement and openness to change values (such as financial success and hedonism) are less emphasized. These shared value patterns across West African countries may reflect common cultural and social factors, lending confidence to the results of this survey given the observed similarities.]

And continued explained in line 486 to 494,

[Numerous studies have shown that when people feel anxious about risks and threats, they tend to emphasize values related to self-preservation, such as tradition and security. Conversely, values associated with openness to change, such as stimulation and self-direction, often decrease as people adapt to new situations. In this study, a comparison of 2018 and 2022 data revealed no significant changes in conservation values. This indicates that value changes among urban Senegalese people deviate from the trends observed in previous research. Specifically, during the global crisis, urban Senegalese did not significantly increase their conservative values. However, self-direction and hedonism—values related to openness to change—decreased significantly.]

In line 554 to 555,

[According to the world Bank, this shift toward cheaper meals is a significant trend in Senegal, aligning with our findings.]

We add In line 591 to 593,

[It is imperative to further investigate whether the value changes observed in this study are temporary or lasting, considering factors such as regional and cultural differences, personal experiences with global crises, and socioeconomic conditions.]

**CONCLUSION**
**Comment 11:** Conclusions: ate least 2 pages, modify and transform into an academic one

**RESPONSE**: : We sincerely appreciate your comments. Actually, the previous manuscript had more than two pages. In the revised version. We accordingly, modify the explanation into academically.

In line 601 to 606,

[Previous research indicates that during crises, people tend to place greater emphasis on conservative values and focus more on social cohesion. However, this study found a different trend among respondents in Senegal. Contrary to expectations, there was no increase in the importance of conservation values typically reported in previous studies. Instead, urban residents in Senegal gave more importance to values such as power and achievement in 2022—values that were considered least important in 2018. ]

In line 655 to 665, we have explained regarding the limitation of this study and future study. Also we add subtitle as Limitations.

[Although the present study reveals important findings, it is important to acknowledge its limitations. First, this study focuses specifically on Saint-Louis, Senegal, limiting its external validity. Future studies should examine whether these findings apply to other regions. Furthermore, due to financial limitations, the sample size is relatively modest compared to larger surveys conducted by international organizations. As a result, the study may not have fully captured the extent of the changes that occurred. Furthermore, the selection methods for the first and second surveys differed, which may affect the comparability of the data.

This study presents a straightforward comparison between two distinct time points, so the impacts of global crises, such as the COVID-19 pandemic and the Russia-Ukraine conflict, are difficult to isolate. It is not possible to identify the individual effects of the various factors that occurred during this period; they can only be presented as hypotheses.]

**ADDITIONAL CHANGE:**

(i)In view of the comments (1) and (3) which led to an expansion of this study and the subsequent discussion of the paper, it has been decided to amend the title of the study in order to provide greater clarity to the argument presented.

New title is [ Exploring shifts in values among urban Senegalese: The impact of global crises on social and cultural norms]

(ii)In order to better organize the discussion in this paper, the title of the subsection has been changed and new subsections have been added as follows;

In line 107, changed sub-section from [Change in value] to [Human values]

In line 125, changed sub-section from [Humen value] to [content of values]

In line 143, changed sub-section from [Impact of series of social change on people’s values] to [Impact of social change on people’s values]

In line 271, new sub-section as [Preferences and data analysis]

In line 442, new sub-section as [Summary]

(iii) In this 2^nd^-revised version of the manuscript, we have rewritten the entire piece in a more academic style in response to the comments received.

**Journal Requirements:** Please review your reference list to ensure that it is complete and correct. If you have cited papers that have been retracted, please include the rationale for doing so in the manuscript text, or remove these references and replace them with relevant current references. Any changes to the reference list should be mentioned in the rebuttal letter that accompanies your revised manuscript. If you need to cite a retracted article, indicate the article’s retracted status in the References list and also include a citation and full reference for the retraction notice.

**Response:** We sincerely appreciate your comments. We have thoroughly reviewed our reference list to ensure its completeness and accuracy.

Thank you for your considerations.
